# Supplementary figures and images for: ZBTB38 is dispensable for antibody responses
Source: PLoS One. 2020 Sep 21;15(9):e0235183. doi: 10.1371/journal.pone.0235183 (PMC7505459; doi:10.1371/journal.pone.0235183)

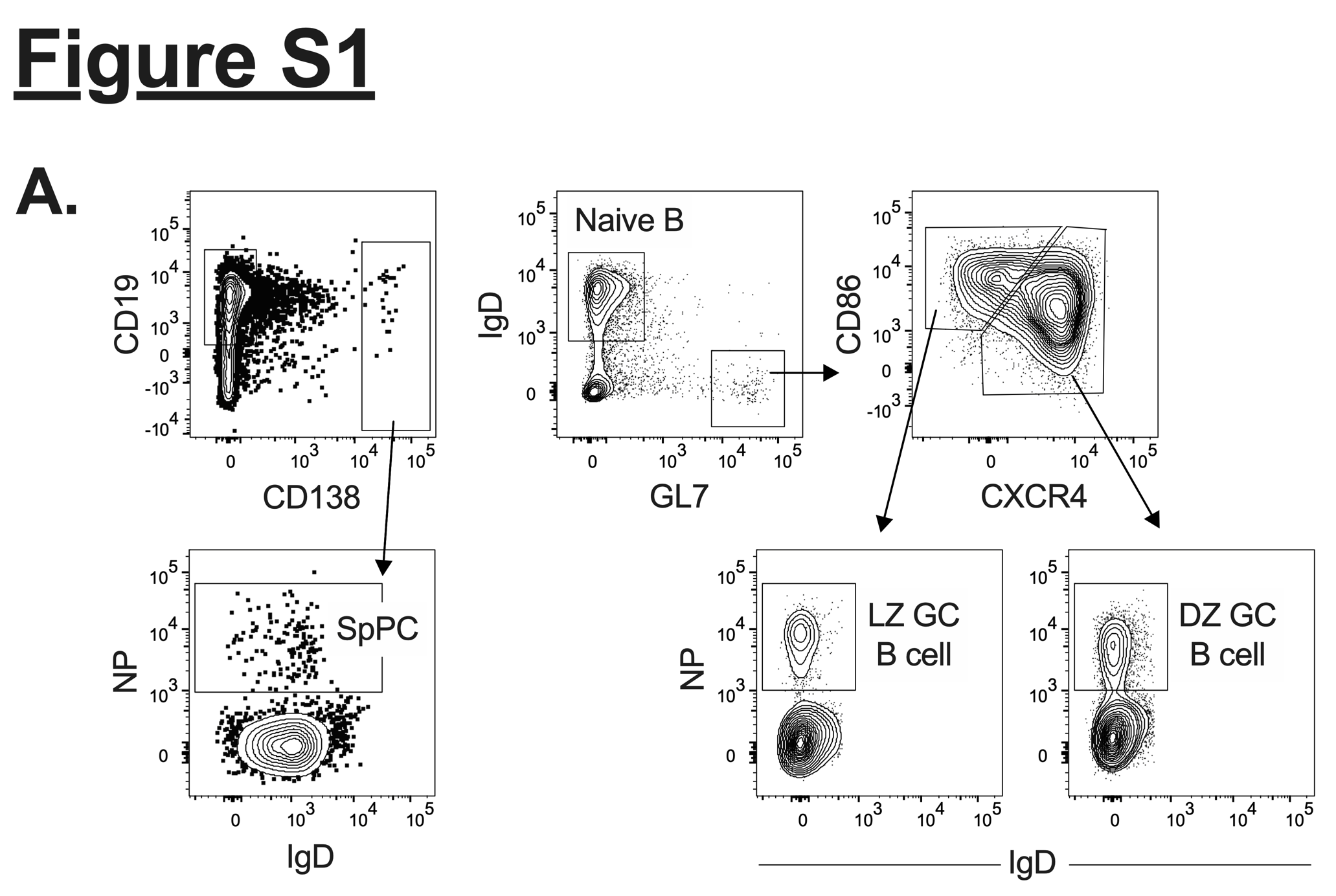

Supplement: S1 Fig — Flow cytometric gating strategies for NP-specific splenic plasma cell (SpPC), light zone (LZ) and dark zone (DZ) germinal center (GC) B cells shown in Fig 1B. (TIFF) [file pone.0235183.s001.tiff]

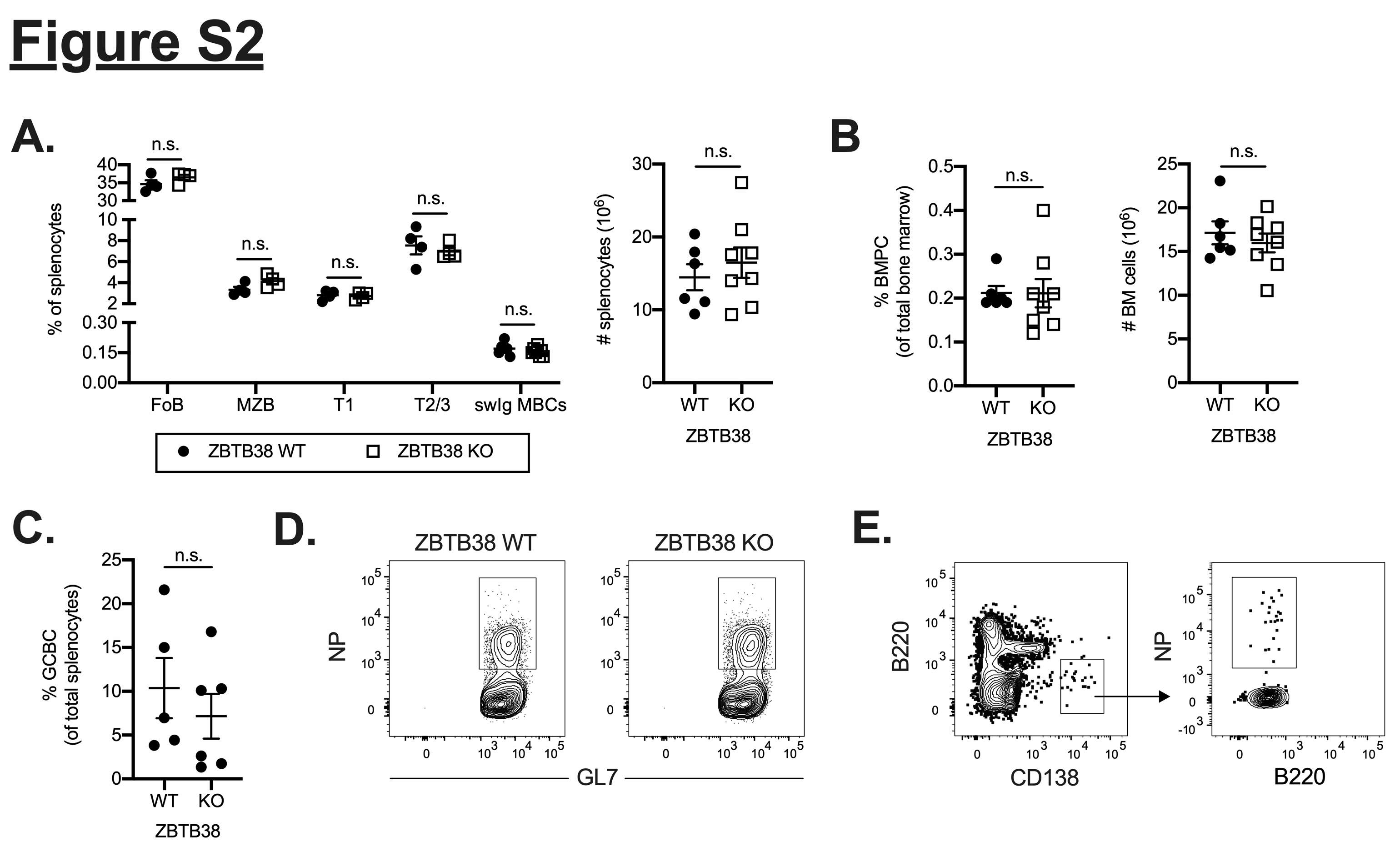

Supplement: S2 Fig — (A) Frequencies of follicular (FoB, CD11b-CD19+IgD+IgM+), marginal zone (MZ, CD11b-CD19+CD93-CD21+CD23-), T1 transitional (CD11b-CD19+CD93+,CD23-), T2/3 transitional (CD11b-CD19+CD93+,CD23+), isotype-switched memory B cells (swIg MBCs, CD19+IgM-IgD-CD80+CCR6+), and bone marrow plasma cells (B220-CD138+) at steady state for ZBTB38 WT and ZBTB38 KO mice. Total numbers of splenocytes are shown in the right panel. Mean ± SEM are shown; each symbol represents an individual mouse. Statistical significance was calculated by Mann-Whitney test; n.s. = not significant (p > 0.05). (B) Frequencies of bone marrow plasma cells (left panel) and total numbers of bone marrow cells (right panel) are shown across genotypes. Statistical significance was calculated by Mann-Whitney test; n.s. = not significant (p > 0.05). (C) Germinal center (CD19+ GL7+) frequency after magnetic bead enrichment for GL7-expressing splenocytes. Statistical significance was calculated by Mann-Whitney test; n.s. = not significant (p > 0.05). (D) Concatenated flow plots of NP+ germinal center B cells (CD19+GL7+IgD-) for ZBTB38 WT (n = 3) and ZBTB38 KO (n = 2) mice. (E) Flow cytometric gating strategy for NP-specific long-lived plasma cells (LLPCs) in the bone marrow. (TIFF) [file pone.0235183.s002.tiff]

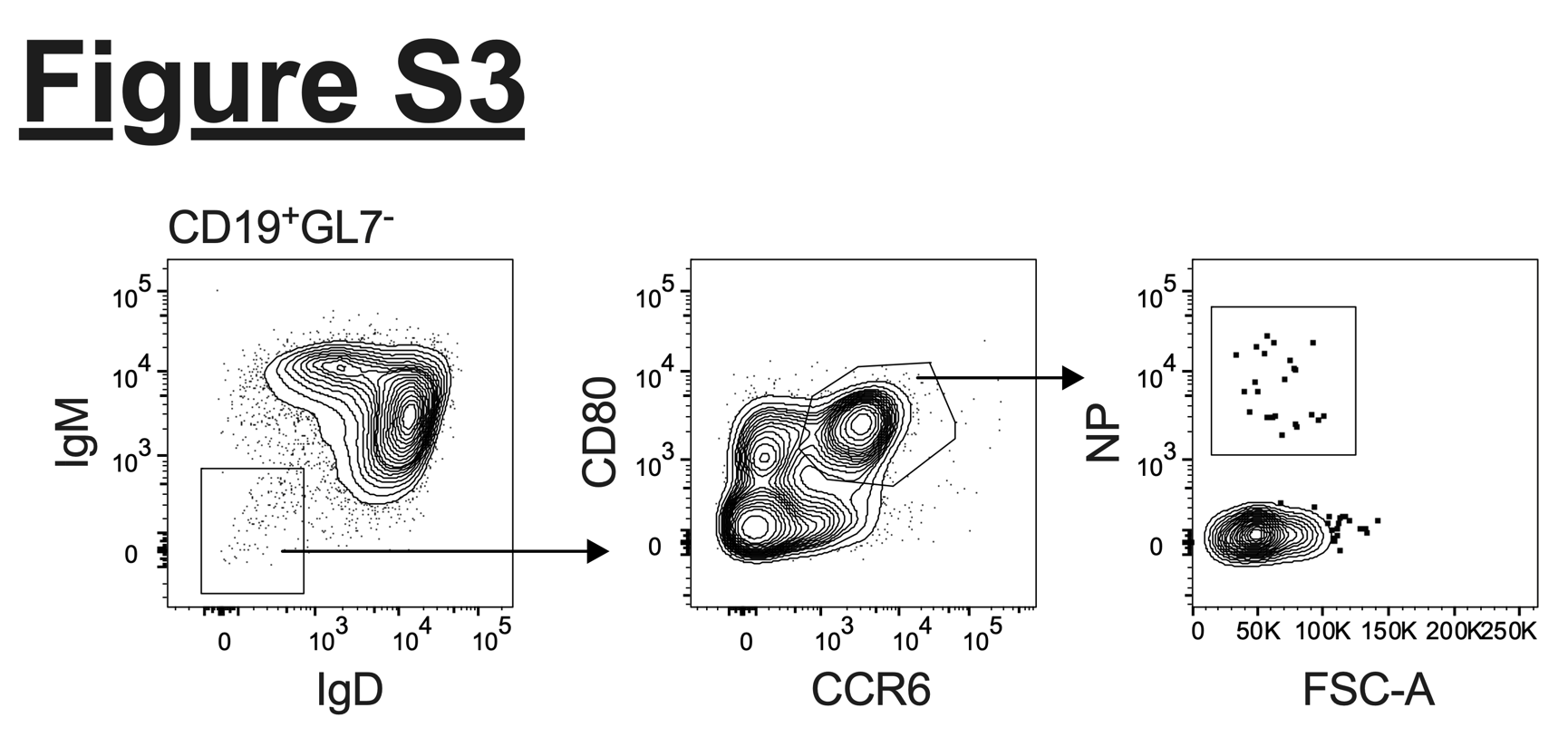

Supplement: S3 Fig — Flow cytometric gating strategy for NP-specific, isotype-switched memory B cells (swIg MBCs) in the spleen. Cells were gated on CD19+GL7-. (TIFF) [file pone.0235183.s003.tiff]

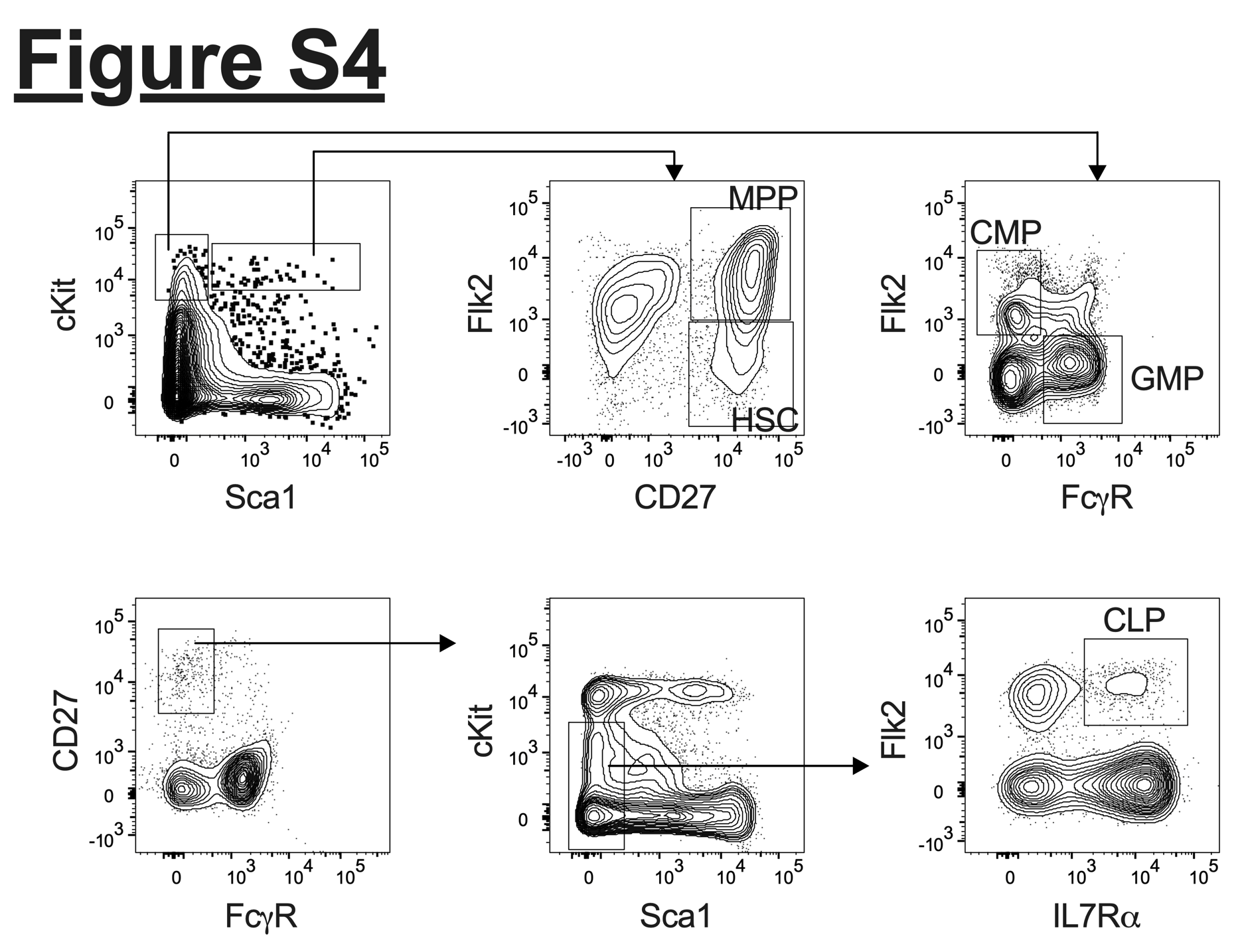

Supplement: S4 Fig — Flow cytometric gating strategies for bone marrow progenitors shown in Fig 6A. HSC, hematopoietic stem cell; MPP, multi-potent progenitor; CMP, common myeloid progenitor; GMP, granulocyte monocyte progenitor; CLP, common lymphoid progenitor. (TIFF) [file pone.0235183.s004.tiff]
