# Supplementary material for: ZBTB38 is dispensable for antibody responses
Source: PLoS One. 2020 Sep 21;15(9):e0235183. doi: 10.1371/journal.pone.0235183 (PMC7505459; doi:10.1371/journal.pone.0235183)
Supplement: S1 File — (PDF) [file pone.0235183.s005.pdf]

S1: Raw Images

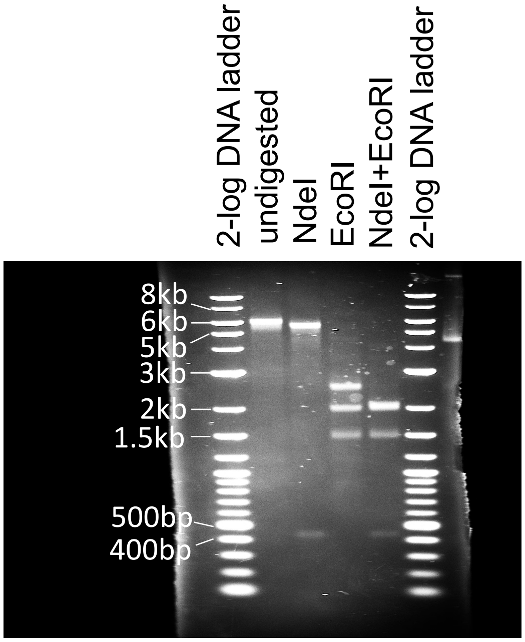

Original DNA gel image used to generate Figure 2B. ProteinSimple AlphaImager Mini was used to visualize the gel.

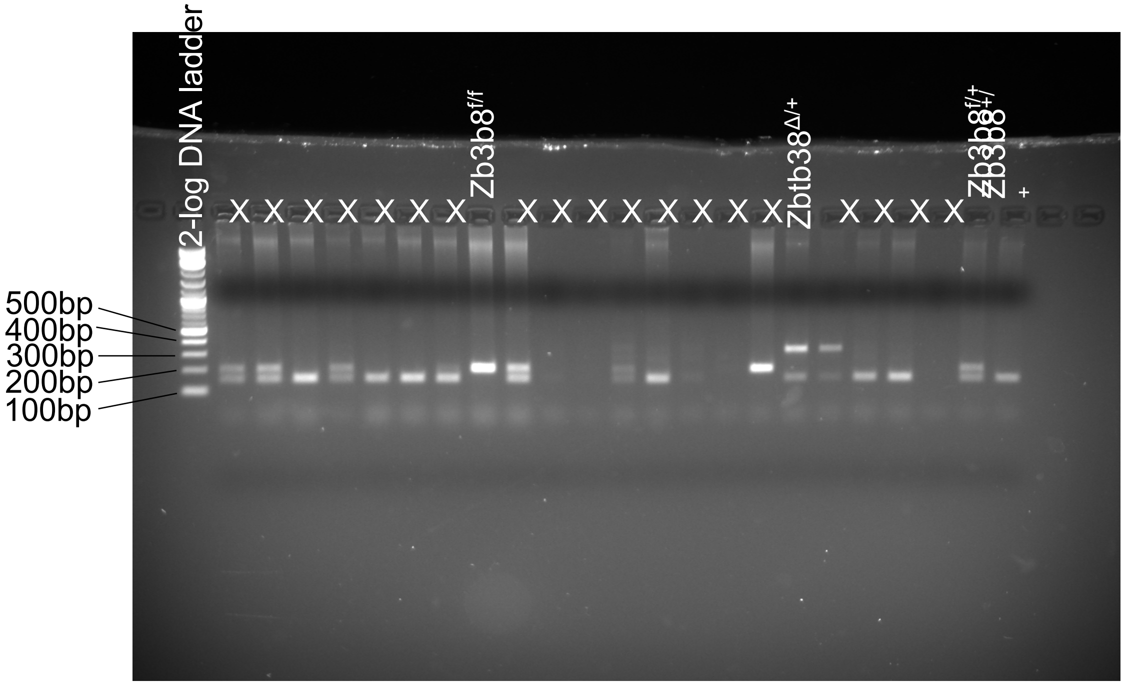

Original DNA gel image used to generate Figure 2C. ProteinSimple AlphaImager Mini was used to visualize the gel.

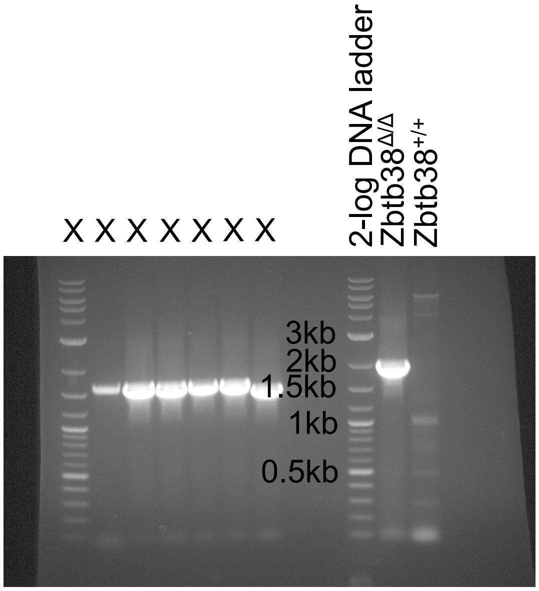

Original DNA gel image used to generate Figure 2D. ProteinSimple AlphaImager Mini was used to visualize the gel.
